# Supplementary material for: Addressing the immigrant screening gap: A protocol for a systematic review on interventions to enhance colorectal cancer screening among immigrants in the United States
Source: PLoS One. 2025 Apr 29;20(4):e0322038. doi: 10.1371/journal.pone.0322038 (PMC12040244; doi:10.1371/journal.pone.0322038)
Supplement: S1 File — (DOCX) [file pone.0322038.s001.docx]

**Table A.** Search String Syntax

| **Database** | **Search string** |
| --- | --- |
| MEDLINE (PubMed) | (((((((((("refugee*"[Title/Abstract]) ) OR ("migrant*"[Title/Abstract])) OR ("emigrants and immigrants"[MeSH Terms])) OR ("immigrant*"[Title/Abstract])) OR ("Transients and Migrants"[MeSH Terms])) OR ("emigrant*"[Title/Abstract])) OR ("immigration"[Title/Abstract])) OR ("foreign born"[Title/Abstract])) AND  ((((((((((((((((((((((("colorectal cancer*"[Title/Abstract]) OR("colorectal neoplasms"[MeSH Terms])) OR ("colorectal neoplasm*"[Title/Abstract])) OR ("neoplasm colorectal"[Title/Abstract])) OR ("colorectal tumors"[Title/Abstract])) OR ("tumor colorectal"[Title/Abstract])) OR ("cancer colorectal"[Title/Abstract])) OR ("colorectal carcinoma*"[Title/Abstract])) OR ("carcinoma* colorectal"[Title/Abstract])) OR ("Colonic Neoplasms"[MeSH Terms])) OR ("colorectal adenocarcinoma*"[Title/Abstract])) OR ("colon cancer*"[Title/Abstract])) OR ("rectum cancer*"[Title/Abstract])) OR ("rectal cancer*"[Title/Abstract])) OR ("adenocarcinoma* colon"[Title/Abstract])) OR ("mucinous adenocarcinoma of the colon"[Title/Abstract])) OR ("signet ring adenocarcinoma of the colon"[Title/Abstract])) OR ("Adenocarcinoma rectum"[Title/Abstract])) OR ("squamous cell carcinoma rectum"[Title/Abstract])) OR ("squamous cell carcinoma rectal"[Title/Abstract])) OR ("Adenocarcinoma rectal"[Title/Abstract])) OR ("cancer of rectum"[Title/Abstract])) OR ("cancer of colon"[Title/Abstract]))) AND  ((((((((((((((((((((((((((((((((((((((((((((((((("colonoscopy*"[Title/Abstract]) OR ("colonoscopy"[MeSH Terms]))) OR ("colonoscopies"[Title/Abstract])) OR ("Colonoscopic Surgical Procedures"[Title/Abstract])) OR ("Colonoscopic Surgical Procedures"[Title/Abstract])) OR ("Procedure Colonoscopic Surgical"[Title/Abstract])) OR ("Procedures Colonoscopic Surgical"[Title/Abstract])) OR ("Surgical Procedure Colonoscopic"[Title/Abstract])) OR ("Surgery, Colonoscopic"[Title/Abstract])) OR ("Surgical Procedures, Colonoscopic"[Title/Abstract])) OR ("Colonoscopic Surgery"[Title/Abstract])) OR ("Colonoscopic Surgeries"[Title/Abstract])) OR ("Surgeries, Colonoscopic"[Title/Abstract])) OR ("sigmoidoscopy*"[Title/Abstract])) OR ("sigmoidoscopy"[MeSH Terms])) OR ("Sigmoidoscopies"[Title/Abstract])) OR ("Proctosigmoidoscopy"[Title/Abstract])) OR ("proctosigmoidoscopies"[Title/Abstract])) OR ("Sigmoidoscopic Surgical Procedures"[Title/Abstract])) OR ("Procedure Sigmoidoscopic Surgical"[Title/Abstract])) OR ("Procedures Sigmoidoscopic Surgical"[Title/Abstract])) OR ("Sigmoidoscopic Surgical Procedure"[Title/Abstract])) OR ("Surgical Procedure Sigmoidoscopic"[Title/Abstract])) OR ("Surgery Sigmoidoscopic"[Title/Abstract])) OR ("Surgical Procedures Sigmoidoscopic"[Title/Abstract])) OR ("Sigmoidoscopic Surgery"[Title/Abstract])) OR ("Sigmoidoscopic Surgeries"[Title/Abstract])) OR ("Surgeries Sigmoidoscopic"[Title/Abstract])) OR ("FOBT"[Title/Abstract])) OR ("Fecal occult blood test"[Title/Abstract])) OR ("Occult Blood"[MeSH Terms])) OR ("Stool test"[Title/Abstract])) OR ("Screen*"[Title/Abstract])) OR ("early detect"[Title/Abstract])) OR ("early diagnosis"[Title/Abstract]))) OR ("early detection of cancer"[Title/Abstract])) OR ("screening rates"[Title/Abstract])) OR ("screening rate"[Title/Abstract])) OR ("FIT"[Title/Abstract])) OR ("fecal immunochemical test"[Title/Abstract])) OR ("mt-sDNA"[Title/Abstract])) OR ("multi-target stool DNA test"[Title/Abstract])) OR ("CT colonography"[Title/Abstract])) OR ("Computer tomographic colonography"[MeSH Terms])) OR ("CT colonography"[MeSH Terms])) OR ("Colonography, CT"[MeSH Terms])) OR ("Virtual colonoscopy"[MeSH Terms])) OR ("Colonoscopy, Virtual"[MeSH Terms]) |
| Cochrane Library (Wiley) | ((((((((((refugee*:ti,ab)) OR (migrant*:ti,ab)) OR ([mh "emigrants and immigrants"])) OR (immigrant*:ti,ab)) OR ([mh "Transients and Migrants"])) OR (emigrant*:ti,ab)) OR (immigration:ti,ab)) OR ("foreign born":ti,ab)) AND (((((((((((((((((((((((("colorectal" NEXT cancer*):ti,ab) OR ([mh "colorectal neoplasms"])) OR (("colorectal" NEXT neoplasm*):ti,ab)) OR ("neoplasm colorectal":ti,ab)) OR ("colorectal tumors":ti,ab)) OR ("tumor colorectal":ti,ab)) OR ("cancer colorectal":ti,ab)) OR (("colorectal" NEXT carcinoma*):ti,ab)) OR ((carcinoma* NEXT "colorectal"):ti,ab)) OR ([mh "Colonic Neoplasms"])) OR (("colorectal" NEXT adenocarcinoma*):ti,ab)) OR (("colon" NEXT cancer*):ti,ab)) OR (("rectum" NEXT cancer*):ti,ab)) OR (("rectal" NEXT cancer*):ti,ab)) OR ((adenocarcinoma* NEXT "colon"):ti,ab)) OR ("mucinous adenocarcinoma of the colon":ti,ab)) OR ("signet ring adenocarcinoma of the colon":ti,ab)) OR ("Adenocarcinoma rectum":ti,ab)) OR ("squamous cell carcinoma rectum":ti,ab)) OR ("squamous cell carcinoma rectal":ti,ab)) OR ("Adenocarcinoma rectal":ti,ab)) OR ("cancer of rectum":ti,ab)) OR ("cancer of colon":ti,ab))) AND (((((((((((((((((((((((((((((((((((((((((((((((((colonoscopy*:ti,ab) OR ([mh colonoscopy]))) OR (colonoscopies:ti,ab)) OR ("Colonoscopic Surgical Procedures":ti,ab)) OR ("Colonoscopic Surgical Procedures":ti,ab)) OR ("Procedure Colonoscopic Surgical":ti,ab)) OR ("Procedures Colonoscopic Surgical":ti,ab)) OR ("Surgical Procedure Colonoscopic":ti,ab)) OR ("Surgery, Colonoscopic":ti,ab)) OR ("Surgical Procedures, Colonoscopic":ti,ab)) OR ("Colonoscopic Surgery":ti,ab)) OR ("Colonoscopic Surgeries":ti,ab)) OR ("Surgeries, Colonoscopic":ti,ab)) OR (sigmoidoscopy*:ti,ab)) OR ([mh sigmoidoscopy])) OR (Sigmoidoscopies:ti,ab)) OR (Proctosigmoidoscopy:ti,ab)) OR (proctosigmoidoscopies:ti,ab)) OR ("Sigmoidoscopic Surgical Procedures":ti,ab)) OR ("Procedure Sigmoidoscopic Surgical":ti,ab)) OR ("Procedures Sigmoidoscopic Surgical":ti,ab)) OR ("Sigmoidoscopic Surgical Procedure":ti,ab)) OR ("Surgical Procedure Sigmoidoscopic":ti,ab)) OR ("Surgery Sigmoidoscopic":ti,ab)) OR ("Surgical Procedures Sigmoidoscopic":ti,ab)) OR ("Sigmoidoscopic Surgery":ti,ab)) OR ("Sigmoidoscopic Surgeries":ti,ab)) OR ("Surgeries Sigmoidoscopic":ti,ab)) OR (FOBT:ti,ab)) OR ("Fecal occult blood test":ti,ab)) OR ([mh "Occult Blood"])) OR ("Stool test":ti,ab)) OR (Screen*:ti,ab)) OR ("early detect":ti,ab)) OR ("early diagnosis":ti,ab))) OR ("early detection of cancer":ti,ab)) OR ("screening rates":ti,ab)) OR ("screening rate":ti,ab)) OR (FIT:ti,ab)) OR ("fecal immunochemical test":ti,ab)) OR (mt-sDNA:ti,ab)) OR ("multi-target stool DNA test":ti,ab)) OR ("CT colonography":ti,ab)) OR ([mh "Computer tomographic colonography"])) OR ([mh "CT colonography"])) OR ([mh "Colonography, CT"])) OR ([mh "Virtual colonoscopy"])) OR ([mh "Colonoscopy, Virtual"]) |
| Scopus (Elsevier) | ((((((((((TITLE-ABS(refugee*))) OR (TITLE-ABS(migrant*))) OR (INDEXTERMS("emigrants and immigrants"))) OR (TITLE-ABS(immigrant*))) OR (INDEXTERMS("Transients and Migrants"))) OR (TITLE-ABS(emigrant*))) OR (TITLE-ABS(immigration))) OR (TITLE-ABS("foreign born"))) AND (((((((((((((((((((((((TITLE-ABS("colorectal cancer*")) OR (INDEXTERMS("colorectal neoplasms"))) OR (TITLE-ABS("colorectal neoplasm*"))) OR (TITLE-ABS("neoplasm colorectal"))) OR (TITLE-ABS("colorectal tumors"))) OR (TITLE-ABS("tumor colorectal"))) OR (TITLE-ABS("cancer colorectal"))) OR (TITLE-ABS("colorectal carcinoma*"))) OR (TITLE-ABS("carcinoma* colorectal"))) OR (INDEXTERMS("Colonic Neoplasms"))) OR (TITLE-ABS("colorectal adenocarcinoma*"))) OR (TITLE-ABS("colon cancer*"))) OR (TITLE-ABS("rectum cancer*"))) OR (TITLE-ABS("rectal cancer*"))) OR (TITLE-ABS("adenocarcinoma* colon"))) OR (TITLE-ABS("mucinous adenocarcinoma of the colon"))) OR (TITLE-ABS("signet ring adenocarcinoma of the colon"))) OR (TITLE-ABS("Adenocarcinoma rectum"))) OR (TITLE-ABS("squamous cell carcinoma rectum"))) OR (TITLE-ABS("squamous cell carcinoma rectal"))) OR (TITLE-ABS("Adenocarcinoma rectal"))) OR (TITLE-ABS("cancer of rectum"))) OR (TITLE-ABS("cancer of colon")))) AND  (((((((((((((((((((((((((((((((((((((((((((((((((TITLE-ABS(colonoscopy*)) OR (INDEXTERMS(colonoscopy)))) OR (TITLE-ABS(colonoscopies))) OR (TITLE-ABS("Colonoscopic Surgical Procedures"))) OR (TITLE-ABS("Colonoscopic Surgical Procedures"))) OR (TITLE-ABS("Procedure Colonoscopic Surgical"))) OR (TITLE-ABS("Procedures Colonoscopic Surgical"))) OR (TITLE-ABS("Surgical Procedure Colonoscopic"))) OR (TITLE-ABS("Surgery, Colonoscopic"))) OR (TITLE-ABS("Surgical Procedures, Colonoscopic"))) OR (TITLE-ABS("Colonoscopic Surgery"))) OR (TITLE-ABS("Colonoscopic Surgeries"))) OR (TITLE-ABS("Surgeries, Colonoscopic"))) OR (TITLE-ABS(sigmoidoscopy*))) OR (INDEXTERMS(sigmoidoscopy))) OR (TITLE-ABS(Sigmoidoscopies))) OR (TITLE-ABS(Proctosigmoidoscopy))) OR (TITLE-ABS(proctosigmoidoscopies))) OR (TITLE-ABS("Sigmoidoscopic Surgical Procedures"))) OR (TITLE-ABS("Procedure Sigmoidoscopic Surgical"))) OR (TITLE-ABS("Procedures Sigmoidoscopic Surgical"))) OR (TITLE-ABS("Sigmoidoscopic Surgical Procedure"))) OR (TITLE-ABS("Surgical Procedure Sigmoidoscopic"))) OR (TITLE-ABS("Surgery Sigmoidoscopic"))) OR (TITLE-ABS("Surgical Procedures Sigmoidoscopic"))) OR (TITLE-ABS("Sigmoidoscopic Surgery"))) OR (TITLE-ABS("Sigmoidoscopic Surgeries"))) OR (TITLE-ABS("Surgeries Sigmoidoscopic"))) OR (TITLE-ABS(FOBT))) OR (TITLE-ABS("Fecal occult blood test"))) OR (INDEXTERMS("Occult Blood"))) OR (TITLE-ABS("Stool test"))) OR (TITLE-ABS(Screen*))) OR (TITLE-ABS("early detect"))) OR (TITLE-ABS("early diagnosis")))) OR (TITLE-ABS("early detection of cancer"))) OR (TITLE-ABS("screening rates"))) OR (TITLE-ABS("screening rate"))) OR (TITLE-ABS(FIT))) OR (TITLE-ABS("fecal immunochemical test"))) OR (TITLE-ABS(mt-sDNA))) OR (TITLE-ABS("multi-target stool DNA test"))) OR (TITLE-ABS("CT colonography"))) OR (INDEXTERMS("Computer tomographic colonography"))) OR (INDEXTERMS("CT colonography"))) OR (INDEXTERMS("Colonography, CT"))) OR (INDEXTERMS("Virtual colonoscopy"))) OR (INDEXTERMS("Colonoscopy, Virtual")) |
| Embase (Ovid) | ((((((((((refugee*.tw.)) OR (migrant*.tw.)) OR (exp "emigrants and immigrants"/)) OR (immigrant*.tw.)) OR (exp "Transients and Migrants"/)) OR (emigrant*.tw.)) OR (immigration.tw.)) OR ("foreign born".tw.)) AND ((((((((((((((((((((((("colorectal cancer*".tw.) OR (exp "colorectal neoplasms"/)) OR ("colorectal neoplasm*".tw.)) OR ("neoplasm colorectal".tw.)) OR ("colorectal tumors".tw.)) OR ("tumor colorectal".tw.)) OR ("cancer colorectal".tw.)) OR ("colorectal carcinoma*".tw.)) OR ("carcinoma* colorectal".tw.)) OR (exp "Colonic Neoplasms"/)) OR ("colorectal adenocarcinoma*".tw.)) OR ("colon cancer*".tw.)) OR ("rectum cancer*".tw.)) OR ("rectal cancer*".tw.)) OR ("adenocarcinoma* colon".tw.)) OR ("mucinous adenocarcinoma of the colon".tw.)) OR ("signet ring adenocarcinoma of the colon".tw.)) OR ("Adenocarcinoma rectum".tw.)) OR ("squamous cell carcinoma rectum".tw.)) OR ("squamous cell carcinoma rectal".tw.)) OR ("Adenocarcinoma rectal".tw.)) OR ("cancer of rectum".tw.)) OR ("cancer of colon".tw.))) AND (((((((((((((((((((((((((((((((((((((((((((((((((colonoscopy*.tw.) OR (exp colonoscopy/))) OR (colonoscopies.tw.)) OR ("Colonoscopic Surgical Procedures".tw.)) OR ("Colonoscopic Surgical Procedures".tw.)) OR ("Procedure Colonoscopic Surgical".tw.)) OR ("Procedures Colonoscopic Surgical".tw.)) OR ("Surgical Procedure Colonoscopic".tw.)) OR ("Surgery, Colonoscopic".tw.)) OR ("Surgical Procedures, Colonoscopic".tw.)) OR ("Colonoscopic Surgery".tw.)) OR ("Colonoscopic Surgeries".tw.)) OR ("Surgeries, Colonoscopic".tw.)) OR (sigmoidoscopy*.tw.)) OR (exp sigmoidoscopy/)) OR (Sigmoidoscopies.tw.)) OR (Proctosigmoidoscopy.tw.)) OR (proctosigmoidoscopies.tw.)) OR ("Sigmoidoscopic Surgical Procedures".tw.)) OR ("Procedure Sigmoidoscopic Surgical".tw.)) OR ("Procedures Sigmoidoscopic Surgical".tw.)) OR ("Sigmoidoscopic Surgical Procedure".tw.)) OR ("Surgical Procedure Sigmoidoscopic".tw.)) OR ("Surgery Sigmoidoscopic".tw.)) OR ("Surgical Procedures Sigmoidoscopic".tw.)) OR ("Sigmoidoscopic Surgery".tw.)) OR ("Sigmoidoscopic Surgeries".tw.)) OR ("Surgeries Sigmoidoscopic".tw.)) OR (FOBT.tw.)) OR ("Fecal occult blood test".tw.)) OR (exp "Occult Blood"/)) OR ("Stool test".tw.)) OR (Screen*.tw.)) OR ("early detect".tw.)) OR ("early diagnosis".tw.))) OR ("early detection of cancer".tw.)) OR ("screening rates".tw.)) OR ("screening rate".tw.)) OR (FIT.tw.)) OR ("fecal immunochemical test".tw.)) OR (mt-sDNA.tw.)) OR ("multi-target stool DNA test".tw.)) OR ("CT colonography".tw.)) OR (exp "Computer tomographic colonography"/)) OR (exp "CT colonography"/)) OR (exp "Colonography, CT"/)) OR (exp "Virtual colonoscopy"/)) OR (exp "Colonoscopy, Virtual"/) |
| CINAHL (Ebsco): | (((((((((((TI refugee* OR AB refugee*))) OR ((TI migrant* OR AB migrant*))) OR ((MH "emigrants and immigrants+"))) OR ((TI immigrant* OR AB immigrant*))) OR ((MH "Transients and Migrants+"))) OR ((TI emigrant* OR AB emigrant*))) OR ((TI immigration OR AB immigration))) OR ((TI "foreign born" OR AB "foreign born"))) AND ((((((((((((((((((((((((TI "colorectal cancer*" OR AB "colorectal cancer*")) OR ((MH "colorectal neoplasms+"))) OR ((TI "colorectal neoplasm*" OR AB "colorectal neoplasm*"))) OR ((TI "neoplasm colorectal" OR AB "neoplasm colorectal"))) OR ((TI "colorectal tumors" OR AB "colorectal tumors"))) OR ((TI "tumor colorectal" OR AB "tumor colorectal"))) OR ((TI "cancer colorectal" OR AB "cancer colorectal"))) OR ((TI "colorectal carcinoma*" OR AB "colorectal carcinoma*"))) OR ((TI "carcinoma* colorectal" OR AB "carcinoma* colorectal"))) OR ((MH "Colonic Neoplasms+"))) OR ((TI "colorectal adenocarcinoma*" OR AB "colorectal adenocarcinoma*"))) OR ((TI "colon cancer*" OR AB "colon cancer*"))) OR ((TI "rectum cancer*" OR AB "rectum cancer*"))) OR ((TI "rectal cancer*" OR AB "rectal cancer*"))) OR ((TI "adenocarcinoma* colon" OR AB "adenocarcinoma* colon"))) OR ((TI "mucinous adenocarcinoma of the colon" OR AB "mucinous adenocarcinoma of the colon"))) OR ((TI "signet ring adenocarcinoma of the colon" OR AB "signet ring adenocarcinoma of the colon"))) OR ((TI "Adenocarcinoma rectum" OR AB "Adenocarcinoma rectum"))) OR ((TI "squamous cell carcinoma rectum" OR AB "squamous cell carcinoma rectum"))) OR ((TI "squamous cell carcinoma rectal" OR AB "squamous cell carcinoma rectal"))) OR ((TI "Adenocarcinoma rectal" OR AB "Adenocarcinoma rectal"))) OR ((TI "cancer of rectum" OR AB "cancer of rectum"))) OR ((TI "cancer of colon" OR AB "cancer of colon")))) AND ((((((((((((((((((((((((((((((((((((((((((((((((((TI colonoscopy* OR AB colonoscopy*)) OR ((MH colonoscopy+)))) OR ((TI colonoscopies OR AB colonoscopies))) OR ((TI "Colonoscopic Surgical Procedures" OR AB "Colonoscopic Surgical Procedures"))) OR ((TI "Colonoscopic Surgical Procedures" OR AB "Colonoscopic Surgical Procedures"))) OR ((TI "Procedure Colonoscopic Surgical" OR AB "Procedure Colonoscopic Surgical"))) OR ((TI "Procedures Colonoscopic Surgical" OR AB "Procedures Colonoscopic Surgical"))) OR ((TI "Surgical Procedure Colonoscopic" OR AB "Surgical Procedure Colonoscopic"))) OR ((TI "Surgery, Colonoscopic" OR AB "Surgery, Colonoscopic"))) OR ((TI "Surgical Procedures, Colonoscopic" OR AB "Surgical Procedures, Colonoscopic"))) OR ((TI "Colonoscopic Surgery" OR AB "Colonoscopic Surgery"))) OR ((TI "Colonoscopic Surgeries" OR AB "Colonoscopic Surgeries"))) OR ((TI "Surgeries, Colonoscopic" OR AB "Surgeries, Colonoscopic"))) OR ((TI sigmoidoscopy* OR AB sigmoidoscopy*))) OR ((MH sigmoidoscopy+))) OR ((TI Sigmoidoscopies OR AB Sigmoidoscopies))) OR ((TI Proctosigmoidoscopy OR AB Proctosigmoidoscopy))) OR ((TI proctosigmoidoscopies OR AB proctosigmoidoscopies))) OR ((TI "Sigmoidoscopic Surgical Procedures" OR AB "Sigmoidoscopic Surgical Procedures"))) OR ((TI "Procedure Sigmoidoscopic Surgical" OR AB "Procedure Sigmoidoscopic Surgical"))) OR ((TI "Procedures Sigmoidoscopic Surgical" OR AB "Procedures Sigmoidoscopic Surgical"))) OR ((TI "Sigmoidoscopic Surgical Procedure" OR AB "Sigmoidoscopic Surgical Procedure"))) OR ((TI "Surgical Procedure Sigmoidoscopic" OR AB "Surgical Procedure Sigmoidoscopic"))) OR ((TI "Surgery Sigmoidoscopic" OR AB "Surgery Sigmoidoscopic"))) OR ((TI "Surgical Procedures Sigmoidoscopic" OR AB "Surgical Procedures Sigmoidoscopic"))) OR ((TI "Sigmoidoscopic Surgery" OR AB "Sigmoidoscopic Surgery"))) OR ((TI "Sigmoidoscopic Surgeries" OR AB "Sigmoidoscopic Surgeries"))) OR ((TI "Surgeries Sigmoidoscopic" OR AB "Surgeries Sigmoidoscopic"))) OR ((TI FOBT OR AB FOBT))) OR ((TI "Fecal occult blood test" OR AB "Fecal occult blood test"))) OR ((MH "Occult Blood+"))) OR ((TI "Stool test" OR AB "Stool test"))) OR ((TI Screen* OR AB Screen*))) OR ((TI "early detect" OR AB "early detect"))) OR ((TI "early diagnosis" OR AB "early diagnosis")))) OR ((TI "early detection of cancer" OR AB "early detection of cancer"))) OR ((TI "screening rates" OR AB "screening rates"))) OR ((TI "screening rate" OR AB "screening rate"))) OR ((TI FIT OR AB FIT))) OR ((TI "fecal immunochemical test" OR AB "fecal immunochemical test"))) OR ((TI mt-sDNA OR AB mt-sDNA))) OR ((TI "multi-target stool DNA test" OR AB "multi-target stool DNA test"))) OR ((TI "CT colonography" OR AB "CT colonography"))) OR ((MH "Computer tomographic colonography+"))) OR ((MH "CT colonography+"))) OR ((MH "Colonography, CT+"))) OR ((MH "Virtual colonoscopy+"))) OR ((MH "Colonoscopy, Virtual+")) |
| Web of Science | ((((((((((refugee*)) OR (migrant*)) OR ("emigrants and immigrants")) OR (immigrant*)) OR ("Transients and Migrants")) OR (emigrant*)) OR (immigration)) OR ("foreign born")) AND ((((((((((((((((((((((("colorectal cancer*") OR ("colorectal neoplasms")) OR ("colorectal neoplasm*")) OR ("neoplasm colorectal")) OR ("colorectal tumors")) OR ("tumor colorectal")) OR ("cancer colorectal")) OR ("colorectal carcinoma*")) OR ("carcinoma* colorectal")) OR ("Colonic Neoplasms")) OR ("colorectal adenocarcinoma*")) OR ("colon cancer*")) OR ("rectum cancer*")) OR ("rectal cancer*")) OR ("adenocarcinoma* colon")) OR ("mucinous adenocarcinoma of the colon")) OR ("signet ring adenocarcinoma of the colon")) OR ("Adenocarcinoma rectum")) OR ("squamous cell carcinoma rectum")) OR ("squamous cell carcinoma rectal")) OR ("Adenocarcinoma rectal")) OR ("cancer of rectum")) OR ("cancer of colon"))) AND (((((((((((((((((((((((((((((((((((((((((((((((((colonoscopy*) OR (colonoscopy))) OR (colonoscopies)) OR ("Colonoscopic Surgical Procedures")) OR ("Colonoscopic Surgical Procedures")) OR ("Procedure Colonoscopic Surgical")) OR ("Procedures Colonoscopic Surgical")) OR ("Surgical Procedure Colonoscopic")) OR ("Surgery, Colonoscopic")) OR ("Surgical Procedures, Colonoscopic")) OR ("Colonoscopic Surgery")) OR ("Colonoscopic Surgeries")) OR ("Surgeries, Colonoscopic")) OR (sigmoidoscopy*)) OR (sigmoidoscopy)) OR (Sigmoidoscopies)) OR (Proctosigmoidoscopy)) OR (proctosigmoidoscopies)) OR ("Sigmoidoscopic Surgical Procedures")) OR ("Procedure Sigmoidoscopic Surgical")) OR ("Procedures Sigmoidoscopic Surgical")) OR ("Sigmoidoscopic Surgical Procedure")) OR ("Surgical Procedure Sigmoidoscopic")) OR ("Surgery Sigmoidoscopic")) OR ("Surgical Procedures Sigmoidoscopic")) OR ("Sigmoidoscopic Surgery")) OR ("Sigmoidoscopic Surgeries")) OR ("Surgeries Sigmoidoscopic")) OR (FOBT)) OR ("Fecal occult blood test")) OR ("Occult Blood")) OR ("Stool test")) OR (Screen*)) OR ("early detect")) OR ("early diagnosis"))) OR ("early detection of cancer")) OR ("screening rates")) OR ("screening rate")) OR (FIT)) OR ("fecal immunochemical test")) OR (mt-sDNA)) OR ("multi-target stool DNA test")) OR ("CT colonography")) OR ("Computer tomographic colonography")) OR ("CT colonography")) OR ("Colonography, CT")) OR ("Virtual colonoscopy")) OR ("Colonoscopy, Virtual") |
| ClinicalTrials.gov | Note: this is a much simpler search string because of limitations with the search engine.  **Condition or disease**: colorectal cancer  **Intervention/Treatment**: screening |

| **Table B.** Draft Data Extraction Template | | |
| --- | --- | --- |
| # | **Extraction Item** | **Responses** |
| **Extraction Details** | |  |
| 1 | Date of form completion | [Free text response] |
| 2 | Reference Citation | [Free text response] |
| **Citation Details** | |  |
| 3 | Title | [Free text response] |
| 4 | First Author | [Free text response] |
| 5 | Year of Publication | [Free text response] |
| 6 | DOI | [Free text response] |
| 7 | Publication Type | Selection one: Peer-reviewed journal Grey literature  Other |
| **Participants** | |  |
| 8 | Nativity (x-American) | [Free text: Enter qualitative or quantitative description as reported] |
| 9 | Mean years since migrated to the US | [Free text response] |
| 10 | Mean years since migrated to the US Intervention Group | [Free text response] |
| 11 | Mean years since migrated to the US Control Group | [Free text response] |
| 12 | Mean Age | [Free text response] |
| 13 | Mean age of intervention participants | [Free text response] |
| 14 | Mean age of control participants | [Free text response] |
| 15 | Age Eligibility Criteria (Provide range of ages) | [Free text response] |
| 16 | Sex | [Free text response] |
| 17 | Sex of intervention participants | [Free text response] |
| 18 | Sex of control participants | [Free text response] |
| 19 | Preferred Language | [Free text response] |
| 20 | Preferred Language in Intervention Group | [Free text response] |
| 21 | Preferred Language in Control Group | [Free text response] |
| 22 | Citizenship | [Free text response] |
| 23 | Citizenship in Control Group | [Free text response] |
| 24 | Citizenship in Intervention Group | [Free text response] |
| 25 | Race | [Free text response] |
| 26 | Race in Control Group | [Free text response] |
| 27 | Race in Intervention Group | [Free text response] |
| 28 | Ethnicity | [Free text response] |
| 29 | Ethnicity in Control Group | [Free text response] |
| 30 | Ethnicity in Intervention Group | [Free text response] |
| 31 | Health Insurance | [Free text response] |
| 32 | Health Insurance Intervention Group | [Free text response] |
| 33 | Health Insurance Control Group | [Free text response] |
| 34 | Description of Participants (1-2 sentences) | [Free text response] |
| 35 | Colorectal cancer screening history inclusion criteria | [Free text response] |
| **Context** | |  |
| 36 | Geographic location - state | [Free text response] |
| 37 | Community Type | Urban; Rural; Suburban |
| 38 | Study setting where intervention took place | Hospital; church; internal med clinic; community health center; primary care center; mixed settings |
| 39 | Study Type/Design | Select One:  Randomized Controlled Trial Cohort Study Case-Control Study |
| **Intervention** | |  |
| 40 | Immigrant Sample Size | [Free text] |
| 41 | Type of Intervention | Select all that apply:  Patient Navigation Only; Patient navigation + culturally targeted materials; Risk communication; Mailed outreach with FIT; Mailed outreach with gFOBT; Mailed outreach with incentive; Mailed outreach without FIT/gFOBT; Individualized education; Non-individualized education; Patient navigation; Personal telephone call; telephone call; education video; education video & brochure |
| 42 | Describe the intervention | [Free text] |
| 43 | Method of measuring the outcome | Objective screening rate; self-reported screening rate |
| 44 | Numbers of Participants in Control Arm | [Free text response] |
| 45 | Numbers of Participants in Intervention Arm | [Free text response] |
| 46 | Number of Participants in Intervention Arm 2 | [Free text response] |
| 47 | Effect Measure |  |
| 48 | Variables adjusted for | [Free text response] |
| 49 | Effect Measure Value & Confidence Interval 1 | [Free text response] |
| 50 | Effect Measure Details 1 | [Free text response] |
| 51 | Effect Measure Value & Confidence Interval 2 | [Free text response] |
| 52 | Effect Measure Details 2 | [Free text response] |
| 53 | Effect Measure Value & Confidence Interval 3 | [Free text response] |
| 54 | Effect Measure Details 3 | [Free text response] |
| 55 | Effect Measure Value & Confidence Interval 4 | [Free text response] |
| 56 | Effect Measure Details 4 | [Free text response] |
| 57 | Study period | [Free text response] |
| 58 | Duration of Treatment Period | [Free text response] |
| 59 | Any limitation to flag | [Free text response] |
| 60 | Screening test | Select all that apply:  Colonscopy; FOBT; Sigmoidoscopy; colonoscopy and FOBT; colonoscopy and sigmoidoscopy; sigmoidoscopy and FOBT; colonoscopy, FOBT, and sigmoidoscopy; unknown |
| 61 | Method of recruitment | Phone; In-person; Flyer; Other |
| 62 | Inclusion criteria | [Free text response] |
| 63 | Exclusion criteria | [Free text response] |
| 64 | Informed consent obtained | Yes; No; Unclear |
| 65 | Power | [Free text response] |
| 66 | % Missing Data | [Free text response] |
| 67 | Partnerships/Collaborations | [Free text response] |
| 68 | Staff/Implementers | [Free text response] |
| 69 | Number of Participants Eligible | [Free text response] |
| 70 | Number of Participants Approached | [Free text response] |
| 71 | Number of Participants Consented | [Free text response] |
| 72 | Number of Participants Declined | [Free text response] |
| 73 | Number of Participants Excluded | [Free text response] |
| 74 | Number of Withdrawals | [Free text response] |
| 75 | Retention Strategies | [Free text response] |
| 76 | Comparator | [Free text response] |
