# Supplementary material for: Addressing the immigrant screening gap: A protocol for a systematic review on interventions to enhance colorectal cancer screening among immigrants in the United States
Source: PLoS One. 2025 Apr 29;20(4):e0322038. doi: 10.1371/journal.pone.0322038 (PMC12040244; doi:10.1371/journal.pone.0322038)
Supplement: Attachment — Submitted filename: Review notes.docx [file pone.0322038.s003.docx]

Review notes/comments:

This is a very solid systematic review protocol. I have one suggestion regarding the data analysis and synthesis plan. Given the expected heterogeneity and the possibility of not conducting a meta-analysis, I recommend that the authors consider incorporating a visual illustration of their qualitative findings using alluvial charts. This method is a powerful way to report overall trends across all studies included in the systematic review. For reference, you may want to look at a recent example of this approach here: [PMC9683573](https://www.ncbi.nlm.nih.gov/pmc/articles/PMC9683573/). <https://www.ncbi.nlm.nih.gov/pmc/articles/PMC9683573/>).
